# Physiological Characteristics of Cotton Subtending Leaf Are Associated With Yield in Contrasting Nitrogen-Efficient Cotton Genotypes

**DOI:** 10.3389/fpls.2022.825116

**Published:** 2022-02-07

**Authors:** Asif Iqbal, Niu Jing, Dong Qiang, Wang Xiangru, Gui Huiping, Zhang Hengheng, Pang Nianchang, Zhang Xiling, Song Meizhen

**Affiliations:** ^1^State Key Laboratory of Cotton Biology, Institute of Cotton Research of Chinese Academy of Agricultural Sciences, Anyang, China; ^2^Western Agricultural Research Center of Chinese Academy of Agricultural Sciences, Changji, China; ^3^Zhengzhou Research Base, State Key Laboratory of Cotton Biology, School of Agricultural Sciences, Zhengzhou University, Zhengzhou, China

**Keywords:** cotton, nitrogen, cotton subtending leaf, photosynthesis, enzymatic activities, yield

## Abstract

Nitrogen (N) plays an important role in various plant physiological processes, but studies on the photosynthetic efficiency and enzymatic activities in the cotton subtending leaves and their contribution to yield are still lacking. This study explored the influence of low, moderate, and high N levels on the growth, photosynthesis, carbon (C) and N metabolizing enzymes, and their contribution to yield in CCRI-69 (N-efficient) and XLZ-30 (N-inefficient). The results showed that moderate to high N levels had significantly improved growth, photosynthesis, and sucrose content of CCRI-69 as compared to XLZ-30. The seed cotton yield and lint yield of CCRI-69 were similar under moderate and high N levels but higher than XLZ-30. Similarly, moderate to high N levels improved the C/N metabolizing enzymatic activities in the subtending leaf of CCRI-69 than XLZ-30. A strong correlation was found between subtending leaf N concentration with C/N metabolizing enzymes, photosynthesis, sucrose contents, boll weight, and seed cotton yield of N-efficient cotton genotype. These findings suggest that subtending leaf N concentration regulates the enzymatic activities and has a key role in improving the yield. These parameters may be considered for breeding N-efficient cotton genotypes, which might help to reduce fertilizer loss and improve crop productivity.

## Introduction

Cotton is an important cash crop grown all over the world, and China is the major producer and consumer (Mao, 2013; China Statistical Yearbook, 2019). Regardless of the introduction of mechanization, high-yielding cultivars, and high input rate, the yield of cotton in China has been stagnant for a couple of decades due to many reasons (Yang et al., 2013; Dai and Dong, 2014). Among the inputs, proper field management like balanced fertilization and the use of nutrient-efficient cotton genotypes may be the limiting factor of poor cotton growth and productivity (Dai and Dong, 2014). Among various essential nutrients, nitrogen (N) is one of the most important nutrients for optimum cotton growth and production (Iqbal et al., 2020a). N participated in various physiological processes crucial for crop production, such as maintaining photosynthetic and sink capacity (Yu et al., 2016). It also plays an important role in cotton growth and is required in large amounts as compared to other nutrients (Hou et al., 2007). The problem of N management in cotton is its indeterminate growth behavior (Chen et al., 2019), which results in imbalanced N fertilization that causes uneven growth (Niu et al., 2021). This inadequate N application leads to a 50% reduction in the yield (Jones et al., 2013; Iqbal et al., 2015). As a result, a huge amount of N fertilizers are applied (Sarasketa et al., 2014) and their application may increase threefold in the coming decades (Good et al., 2004). The consequences of excessive N fertilization are prolonged vegetative growth (Chen et al., 2019), high cost of production (Hou et al., 2007), environmental pollution (Qiao et al., 2012), and lower N use efficiency (NUE) (Miao et al., 2011; Iqbal et al., 2020a). To decrease this costly and important production factor, there is an urgent need to develop/screen cultivars with high NUE (Den Herder et al., 2010; Kant et al., 2010). In our previous studies, we have identified cotton genotypes with differing NUEs and biomass potential at the seedling stage (Zhang et al., 2018; Iqbal et al., 2020a,b). However, there is a lack of knowledge about the genotypic variations in the photosynthetic efficiency, N metabolism of cotton subtending leaves, and their contribution to yield.

Adequate N fertilization is important for optimum plant growth and N metabolism (Iqbal et al., 2020a). Efficient N uptake and utilization is the guarantee of high photosynthesis and its translocation from source to sink tissues. Similarly, amino acids, proteins, and other N-containing compounds are totally dependent on N, and therefore N deficiency leads to imbalance in amino acids (Iqbal et al., 2020a,b). This imbalance in amino acids disturbs protein synthesis and translocation of amino acids to other plant parts. Studies have found that N application significantly enhanced the enzymatic activities related to N metabolism (Krapp et al., 2011; Gu et al., 2013). Nitrate reductase and nitrite reductase are the rate-limiting enzymes responsible for the conversion of nitrate to ammonia (Singh and Usha, 2003). The ammonia produced is then converted into glutamine and glutamate by glutamine synthetase and glutamate synthase, respectively, which are then converted into various amino acids, proteins, and N-containing compounds (Iqbal et al., 2020a). In our previous studies, we have found that the enzymatic activities related to N metabolism have a close relationship with NUE in cotton at the seedling stage (Iqbal et al., 2020a,b). There are many ways to regulate N metabolism like protein degradation, N assimilation, stagnant translocation of amino acids, and its utilization for the TCA cycle (Wang et al., 2012; Hu et al., 2016). Thus, N-efficient genotype and adequate N are important to maintain N metabolism, amino acid and protein production, and its partitioning to other plant parts.

In cotton, the subtending leaf is the basic source of photosynthates for boll development and is the major contributor to cotton yield, especially boll weight (Liu et al., 2013). During boll development, it provides about 60–87% of the photosynthates (Liu et al., 2013). Bolls and their subtending leaves are the sinks and source for photosynthates, and their relationships reflect the coordination between vegetative and reproductive growths and affect cotton yield (Xie et al., 2003). Studies have found that early sink development ability and stronger reproductive growth potential are important characteristics of high-yielding cotton cultivars (Wang et al., 2004). Sucrose and starch are the end products of photosynthesis and are translocated into the sink to provide C and energy for growth and development (Lunn and Hatch, 1995). Moreover, high boll weight and yield could be obtained by strong photosynthesis in the cotton subtending leaf as well as the effective distribution of the photoassimilates to the sinks (Richards, 2000; Wang, 2007). It was suggested that the partitioning of photosynthates and N to the reproductive parts needs to be increased to improve grain size and number (Richards, 2000). Therefore, improvement in cotton yield needs the investigation of photosynthesis in the subtending leaf (Keru et al., 2002) during boll formation, as well as the translocation of the photosynthates to the sink (Pettigrew and Gerik, 2007). Crop yield formation is essentially due to the process of source–sink interaction (Chen et al., 2019). The coordination of the source–sink system in cotton is very important because of its long coexisting period and indeterminate growth behavior (Chen et al., 2019). Any imbalance in the source–sink system will finally affect yield (Gwathmey and Clement, 2010). As mentioned earlier, the boll and its subtending leaves act as source and sink of photosynthesis, respectively, and are closely associated with the synthesis, translocation, and distribution of photosynthates. However, little is known about the photosynthesis, C/N metabolism of the cotton subtending leaf and its relationship with cotton yield.

After a series of experiments, we have identified the high N-efficient cotton genotype (CCRI-69) that can produce high economic yield at a reduced N fertilizer. The genotype CCRI-69 has a flourishing root system, high photosynthetic efficiency, more biomass production, N metabolism, and NUE (Iqbal et al., 2020a). However, the genotypic difference in growth, photosynthetic capacity, and C/N metabolism in the cotton subtending leaf and their relationship with yield are still to be elucidated. Keeping in view the importance of photosynthesis, C/N metabolism in the cotton subtending leaf and its contribution to yield, we hypothesized that genotype CCRI-69 may have high photosynthetic activity, efficient C/N metabolism, and translocation of photosynthates from source to sink tissues to maintain high yield. Subsequently, the objective of our study was to explore the relative performance of cotton genotypes for growth, effective photosynthetic activity, and C/N metabolism in the cotton subtending leaves and their relationship with cotton yield.

## Materials and Methods

### Plant Materials and Experimental Conditions

In the current study, contrasting N-efficient cotton genotypes were used (CRI 69, N-efficient and XLZ 30, N-inefficient). They were selected from our previous phenotypic ranking of 270 cotton genotypes based on biomass and NUE in the pot and hydroponic culture (Gui et al., 2018; Zhang et al., 2018; Iqbal et al., 2020a).

#### Pot Experiment

The pot experiment was carried out in the greenhouse (36^°^06′N, 114^°^21′E) at the experimental farm of the Cotton Research Institute, Chinese Academy of Agricultural Sciences (CRI, CAAS) during 2018 and 2019. The test soil was 0–20 cm low-fertile arable soil, taken from the cotton field of the experimental farm of the CRI, CAAS. The contrasting N-efficient cotton genotypes were grown in a black plastic bucket (height: 33 cm, top diameter: 36 cm, and bottom diameter 30 cm) having 24 kg of soil collected from the farm field. There were 36 replicates for each of the four cultivars and 4 N treatments such as N0 (0 g N kg^–1^ soil), N1 (0.075 g N kg^–1^ soil), N2 (0.150 g N kg^–1^ soil), and N3 (0.225 g N kg^–1^ soil). The experiment consisted of 576 pots with each treatment having 16 pots and one plant per pot. N was applied in the form of urea (46.4% N) in three equal splits i.e., 1/3 basal application, 1/3 topdressing at the seedling stage, 1/3 topdressing at the flowering stage. In addition, phosphorus (P) from superphosphate (45% P_2_O_5_) and potassium (K) from potassium sulfate (50% K_2_O) were applied at the rate of 5.21 g and 5.76 g per pot, respectively. Healthy seeds of each cultivar were manually sown on May 1, 2018, and April 29, 2019. The seedlings were fixed during the seedling stage, and each pot retains a uniform growth till end of the experiment. During the period, the rain-proof canopy was used to block the natural precipitation, and it was opened on sunny days to ensure sufficient and consistent light. Except for different N treatments, other cultivation and management measures remain the same for each pot.

#### Field Experiments

The field experiments were carried out in the experimental farm of the CRI, CAAS, Anyang, Henan during 2018 and 2019. The experimental field has been used for cotton cultivation for the past 5 years. The meteorological data are shown in Supplementary Figure 1. There was no significant difference in annual average temperature; however, a huge difference in rainfall was observed between the 2 years, especially in the early and later stages of plant growth. The total monthly rainfall in the growing season was 12.9, 56, and 45 mm in 2018, 2019, and the during last 5 years, respectively. The 2-year experiment was conducted under a split-plot experimental design with three replications, where the main plot consisted of four N levels, and cotton genotypes were assigned to the subplot. Urea (46% N) was the sole source of applied N at the rate of 0 (N0), 112.5 (N1), 225 (N2: the amount of N applied by local farmers), and 337.5 (N3) kg N ha^–1^. As for N treatment, half of the target N was used as a basal fertilizer and the other half was applied at the full blooming stage. In addition, P and K were applied as basal fertilizers at the rate of 120 kg P ha^–1^ using triple superphosphate (46% P_2_O_5_) and 150 kg K ha^–1^ using potassium sulfate (50% K_2_O). The experimental plot was 6.4 m wide × 8 m long with 8 rows, each spaced 0.8 m apart. A uniform cotton planting density of approximately 52,500 plants per hectare was maintained. The cultivars were planted on April 28, 2018 and May 3, 2019, respectively. The cultivation management methods were consistent for 2 years.

#### Hydroponic Experiment

A greenhouse experiment was conducted at the CRI, CAAS, Anyang, China. Two contrasting N-efficient cotton genotypes (CCRI-69, N-efficient and XLZ-30, N-inefficient) were grown in the hydroponic culture as described by Iqbal et al. (2020a,b). The seeds of both cotton genotypes were planted in a mixture of sands and vermiculite for 7 days in a germinator. After 1 week, the uniform seedlings were selected and transplanted into black color plastic boxes (10 L) in the growth chamber (16/8 light/dark period, 28°C temperature, 60% relative humidity). Plants were supplied with half-strength Hoagland solutions during the first week followed by full-strength Hoagland solution [2 mM KH_2_PO_4_, 2 mM KCL, 2 mM MgSO_4_, 0.1 mM EDTA⋅Fe⋅Na, 46.2 uM H_3_BO_3_, 9.1 uM MnCl_2_⋅4H_2_O, 0.8 uM ZnSO_4_⋅7H_2_O, 0.3 uM CuSO_4_⋅5H_2_O, 1.0 uM (NH_4_)_6_Mo_7_O_24_⋅4H_2_O)] till the end of the experiment (Iqbal et al., 2020a). At two true leaves stage, seedlings of both the cotton genotypes were divided into three categories, low (0.25 mM), moderate (2.5 mM), and high (5 mM) N conditions. The solutions were changed every week till the end of the experiment. Every week, the position of boxes was changed to avoid the position effect.

### Sampling

Plants attaining white flowers were selected, and the subtending leaves at the first position in the fruiting branches were tagged. About 6–8 tagged leaves from each treatment were collected from 9:00 to 10: 00 a.m. on 10, 25, and 40 days post-anthesis (DPA). The collected leaves were divided into two parts, one was directly frozen in the liquid N and then put in a freezer (−80°C) for the measurements of N metabolizing enzymatic activities.

### Plant Morphology

After harvesting, the plants from each treatment were separately divided into root, stem, leaves, and bolls. The parts of root, stem, and leaves were then placed in the oven for 1 h at 105^°^C, followed by 80^°^C for the next 48 h. The dry weight of root, stem, leaves, and bolls was measured on an electronic balance to calculate the dry matter of each tissue. Similarly, the lengths and widths of each leaf were measured and the mean single leaf area was obtained from the product of length, width, and correction factor (0.75) (Iqbal et al., 2020b), and the leaf area plant^–1^ was calculated by multiplying the number of leaves plant^–1^ with mean single leaf area, as described by Iqbal et al. (2020a,b).

### Measurement of Photosynthetic Attributes

Photosynthetic attributes were analyzed from the top three subtending leaves of six randomly selected plants using a portable photosynthesis machine (Li-Cor-6800; Li-Cor, Inc., Lincoln, NE, United States) from 9:00 a.m. to 11:00 a.m. (He et al., 2011) on 10, 25, and 40 DPA. In the growth chamber, the CO_2_ concentration and the light intensity were maintained as suggested by Cao et al. (2012).

### Measurements of Nitrogen Concentrations and N Use Efficiency Traits

The oven-dried samples (root, shoot, and leaf) were ground, and 0.1 g of the samples was taken in digestion tubes for digestion using sulfuric acid (H_2_SO_4_) and hydrogen peroxide (H_2_O_2_). Two milliliters of H_2_SO_4_ was added to the digestion tubes and incubated overnight at room temperature. The next day, H_2_SO_4_ was again added to the tubes and they were rotated. The tubes were placed on a hot plate and heated up to 350°C for 30 min until fumes were produced. One milliliter of H_2_O_2_ was further added and heated for 20 min. The tubes were removed from the hot plate and these steps were repeated until the material became colorless. The extract was filtered and a 50 mL volume was made using distilled water. The digested leaf sample (5 mL) was then used for the determination of N concentration using the Bran + Luebbe Continuous-Flow AutoAnalyzer III (AA3) as described by Li et al. (2006). N utilization efficiency (NUtE) was calculated as the total plant dry weight divided by N concentration (g TDW mg^–1^ N) and N uptake efficiency (NUpE) was calculated as total N accumulation (N concentration x total plant dry matter) divided by root dry weight (mg N g^–1^ root dry matter) (Iqbal et al., 2020b).

### Measurements of Sucrose and Enzyme Related to Carbon and Nitrogen Metabolism

Nitrate reductase (NR: EC 1.7.1.3) activity was measured according to Iqbal et al. (2020a) and expressed as μg N dioxide (NO_2_^–^) h^–1^ g^–1^ fresh weight (FW). Fresh tissue sample (0.2 g) added with 2.0 mL extraction [25.0 mM phosphate buffer (pH 7.5), 5.0 mM cysteine, and EDTA-Na_2_]. It was ground in an ice bath and centrifuged at 8,000 rpm for 10 min at 4°C. Then, 0.4 mL of the supernatant was mixed with 1.6 mL of a mixture (1.2 mL of 0.1 M KNO_3_ phosphate buffer and 0.4 mL of 2.0 mg mL^–1^ NADH solution). The control received 0.4 mL of 0.1 mM phosphate buffer without the NADH solution. Both treatment and control were kept in a 30°C bath for 30 min. Then, 1.0 mL of 1% p-aminobenzene sulfonic acid and 0.2% α-naphthylamine were added to the supernatant, color developed for 20 min, and centrifuged for 5 min at 4,000 rpm. The absorbance was determined by calorimetry at a wavelength of 540 nm.

The determination method for glutamine synthetase (GS; EC 6.3.1.2) enzyme activity was as described by Iqbal et al. (2020a). One unit of GS activity (U) was defined as a 0.01 change in A540 per minute per mL reaction system. Fresh samples (0.2 g) were mixed with 2.0 mL of an extract (0.05 M Tris–HCl buffer, pH 8.0, 2.0 mM MgSO_4_, 2.0 mM DTT, and 0.4 M sucrose), minced in an ice-cold mortar, and centrifuged at 15,000 rpm for 20 min at 4°C. Then, 0.7 mL of the supernatant was mixed with 1.6 mL of 0.1 M Tris–HCL buffer (pH 7.4, 80.0 mM MgSO_4_, 20.0 mM sodium glutamate, 20.0 mM cysteine, 2.0 mM EDTA, containing 80.0 mM HONH_3_Cl) and 0.7 mL of 40.0 mM ATP solution. The mixture was placed in a water bath for 30 min at 25°C, to which 1.0 mL of a chromogenic reagent (0.2 M trichloroacetic acid, 0.37 M FeCl_3_, and 0.6 M HCl) was added, incubated for 15 min, and centrifuged at 5,000 rpm for 10 min at 25°C, then the supernatant was collected and the absorbance was measured at a wavelength of 540 nm. The reaction mixture of 1.6 mL of 0.1 M Tris–HCl solution (pH 7.4, not containing 80.0 mM HONH_3_Cl) was added as control.

Glutamate synthase (GOGAT; EC 1.4.7.1) and glutamate dehydrogenase (GDH; EC 1.4.1.2) activities were determined by spectrophotometer according to the absorbance of NADH at a wavelength of 340 nm (Iqbal et al., 2020a). One unit of GOGAT and one unit of GDH have calculated the oxidation of 1.0 nmol of NADH per min. Sample weighing, adding extract, and centrifugation methods for determining GOGAT and GDH activities were the same as those of GS. Then, 100 mM K^+^-phosphate (pH 7.6), containing 0.1% (v:v) 2-mercaptoethanol, was used to determine the activity of the GDH and GOGAT enzymes. In addition, the reaction solution of GOGAT was 2.5 mM α-ketoglutarate, 100 μM NADH, 10.0 mM L-glutamine, and 1.0 mM aminooxyacetate, and that of GDH was 2.5 mM α-ketoglutarate, 100.0 μM NADH, and 100.0 mM (NH_4_^+^)_2_SO_4_.

Glutamic-oxaloacetic transaminase (GOT; EC 2.6.1.1) and glutamic-pyruvic transaminase (GPT; EC 2.6.1.15) activities were assayed according to the standard protocol. Modest enzyme extract was added to the substrate solution (pH 7.4) containing 2 mM α-oxoglutarate and 200 mM DL-aspartate (GOT) or 200 mM DL-alanine (GPT). The mixture was incubated at 37°C for 1 h, and the reaction was terminated by adding 2, 4-dinitrophenylhydrazine. After incubating the mixture at 37°C again for 20 min, 5 mL 0.4 M NaOH was added and the absorbance of the solution was chromometrically measured at 500 nm. The activities of GOT and GPT were expressed as the generating rate of pyruvic acid per mg protein.

Protease (EC 3.4.21.112) was extracted according to manufacturer protocol. The reaction mixture included 0.4 mL of 0.05 M succinate buffer (containing 10 mM mercaptoethanol, pH 5.5), 0.4 mL of 10 mg/mL azocasein and 0.2 mL of extraction. After 1 h incubation at 35°C, the reaction was terminated by adding 1 mL of 1 N perchloric acid. The mixture was centrifuged at 8,000 × *g* for 15 min and the absorbance of the supernatant was measured at 400 nm.

The sucrose synthase (SS; EC 2.4.1.13) activity was determined by using the commercial chemical kits in accordance with the manufacturer’s instructions provided by Suzhou Comin Biotechnology, Suzhou, China. About 0.1 g fresh leaf sample was homogenized by using liquid nitrogen in pre-chilled mortar and pestle. After halogenation, the sample was extracted with 1 ml extraction buffer (provided by the manufacturer separate for each enzyme) in a 2 ml test tube and centrifuged. The reaction of the enzyme was carried out according to the manual provided by the manufacturer and the absorption values were recorded at A480.

For sucrose phosphate synthase (SPS; EC 2.4.1.14), approximately 1 g of frozen material was ground to a fine powder in an ice bath with 5 mL of 4-(2-hydroxyethyl)-1-piperazineethanesulfonic acid-NaOH buffer (50 mM, pH 7.5) containing 50 mM MgCl_2_, 2 mM EDTA, 0.2% (w/v) bovine serum albumin, and 2% polyvinyl pyrrolidone (PVP). After centrifugation (10,000 × *g*, 10 min), 50 μL of supernatant was mixed with 50 μL of HEPES-NaOH buffer, 20 μL of 50 mM MgCl_2_, 20 μL of 100 mM uridine diphosphoglucose (UDPG), and 20 μL of 100 mM fructose. The mixture was incubated at 30°C for 30 min, and the reaction was stopped by the addition of 200 μL of 2 mM NaOH and boiling for 10 min. The solution was then cooled to room temperature. Next, 1.5 mL of 30% HCl and 0.5 mL of 0.1% resorcin were added and mixed thoroughly. Then, the mixture was incubated at 80°C for 10 min. The solution was cooled to room temperature again and light absorption was measured spectrophotometrically at 480 nm.

Sucrose content was determined by using the commercial chemical kit following the manufacturer’s instructions (Suzhou Comin Biotechnology, Suzhou, China). About 0.1 g dried leaf tissues were extracted with 5 mL of 80% ethanol. The alcoholic extract was centrifuged for 10 min (3,500 × *g*) and then the supernatant was stored at 4°C for further analysis. The determination of the sucrose was assessed according to the protocol provided by the manufacturer. The pellet obtained after centrifugation was dried and then incubated for 48 h at 37°C in buffer acetate (4.5 mM) and α-glucoamylase (0.5%, w/v) and water for further determination. The absorption value of sucrose was recorded at 480 nm. The concentration of sucrose was expressed as mg g^–1^ FW.

### Cotton Yield

Cotton yield and yield attributes were recorded in three harvests and the final yield was attained by adding all the three harvests and presented in g plant^–1^. The number of bolls in each plant was counted and collected to measure boll weight, lint yield, and percentage. Before ginning, bolls were sun-dried to decrease the water content down to 11% (Dong et al., 2004).

### Statistical Analysis

Statistical analysis was performed by using two-way ANOVA in Statistix 10 software. The least significant difference (LSD) was used for mean separation at a 5% level of significance. The figures were drawn with GraphPad Prism 7. All the data results are expressed as mean ± standard error (SE) of three technical and biological replications.

## Results

The results obtained from the hydroponic experiment are explained here, while that of pot and field experiments are only used to know the relationship of subtending leaf N concentration with photosynthesis, sucrose contents, C/N metabolism, boll weight, and yield.

### Plant Growth

**FIGURE 1:**
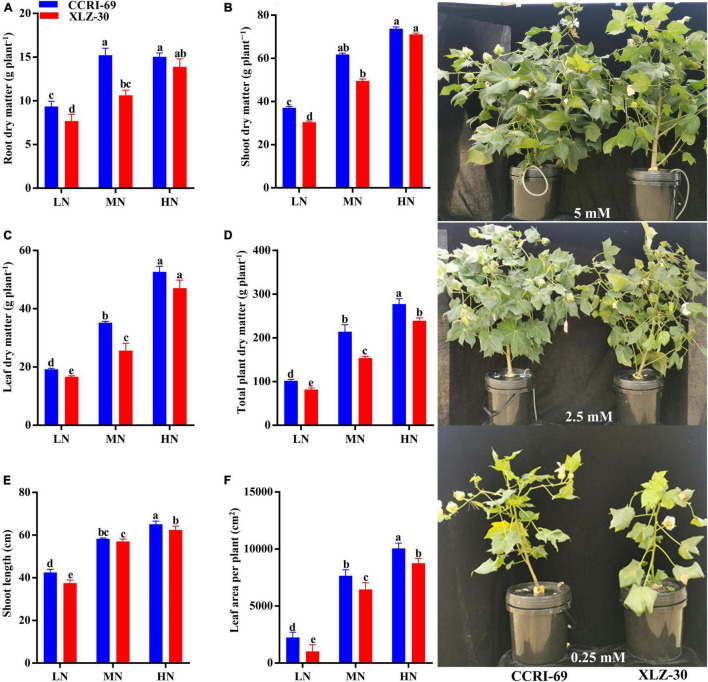
**(A–F)** Root dry matter (g plant^–1^), shoot dry matter (g plant^–1^), leaf dry matter (g plant^–1^), total plant dry matter (g plant^–1^), shoot length (cm), and leaf area per plant (cm^2^), and plant phenotypes of CCRI-69 and XLZ-30 in response to low (LN; 0.25 mM), moderate (MN; 2.5 mM), and high (HN; 5 mM) N levels. Error bars with different small letters show significant differences between genotypes under different N levels (*p* < 0.05).

At the end of the experiment, clear N deficiency symptoms were observed in plants treated with low N level, and these symptoms were more obvious in XLZ-30 as compared to CCRI-69. In both cotton genotypes, a significant increase in growth traits was observed with an increase in N levels, except root and shoot dry matter of CCRI-69 which were similar at moderate and high N levels (Figure 1). In comparison with low N level, shoot length, leaf area plant^–1^, root, shoot, leaf, and total plant dry matter were increased by 37.3, 81.4, 41.1, 53.4, 64.1, and 64.8% under high N levels, respectively (Figure 1). Between the genotypes, CCRI-69 had higher shoot length (5.4%), leaf area plant^–1^ (25.4%), root dry matter (18.7%), shoot dry matter (12.5%), leaf dry matter (16.6%), and total plant dry matter (17.7%) as compared to XLZ-30 (Figure 1). Except for leaf dry matter, other growth traits of XLZ-30 at high N level were statistically similar to that of CCRI-69 at moderate N levels, indicating the higher growth and N efficiency of CCRI-69 as compared to XLZ-30.

### Photosynthetic Attributes of Cotton Subtending Leaf

**FIGURE 2:**
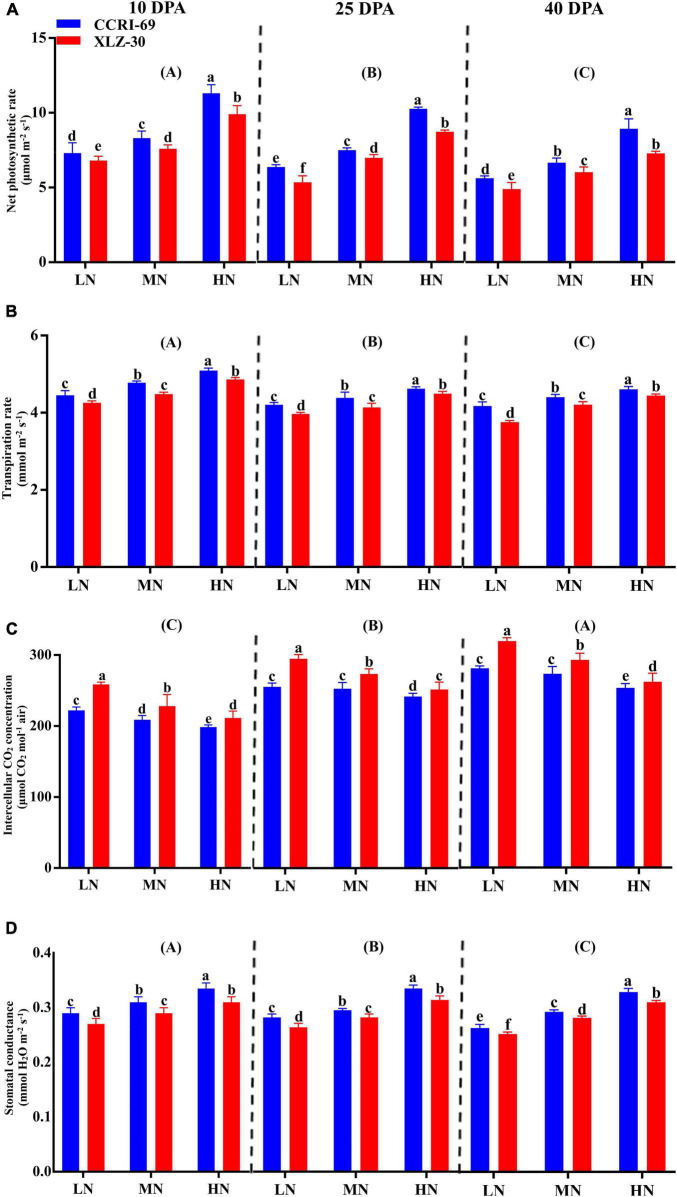
**(A–D)** Net photosynthetic rate (μmol m^–2^ s^–1^), transpiration rate (mmol m^–2^ s^–1^), intercellular CO_2_ concentration (μmol CO_2_ mol^–1^ air), and stomatal conductance (mmol H_2_O m^–2^ s^–1^) of cotton subtending leaves of CCRI-69 and XLZ-30 in response to low (LN; 0.25 mM), moderate (MN; 2.5 mM), and high (HN; 5 mM) N levels at 10, 25, and 40 days post-anthesis (DPA). Error bars with different small letters show significant differences between genotypes under different N levels, and capital letters in the brackets show significant differences among growth stages (10, 25, and 40 DPA) at *p* < 0.05.

Photosynthetic attributes like net photosynthetic rate, transpiration rate, intercellular CO_2_ concentration, and stomatal conductance greatly varied among growth stages (Figure 2). A decreasing trend in all photosynthetic attributes was observed from 10 to 40 days post-anthesis (DPA) except intercellular CO_2_ concentration (Figure 2). Compared to low N level, an increase in net photosynthetic rate (31.9, 32.8, and 33.8%), transpiration rate (9.2, 9.8, and 9.2%), and stomatal conductance (13.6, 15.9, and 15.8%) were found under high N level at 10, 25, and 40 DPA (Figure 2). Irrespective of the N level, CCRI-69 had significantly higher net photosynthetic rate 10.9, 8.4, and 13.5%, transpiration rate 3.8, 4.3, and 4.2%, and stomatal conductance 4.6, 5.8, and 4.1% at 10, 25, and 40 DPA, respectively, as compared to XLZ-30 (Figure 2).

### Enzymatic Activities and Sucrose Content in the Cotton Subtending Leaf

**FIGURE 3:**
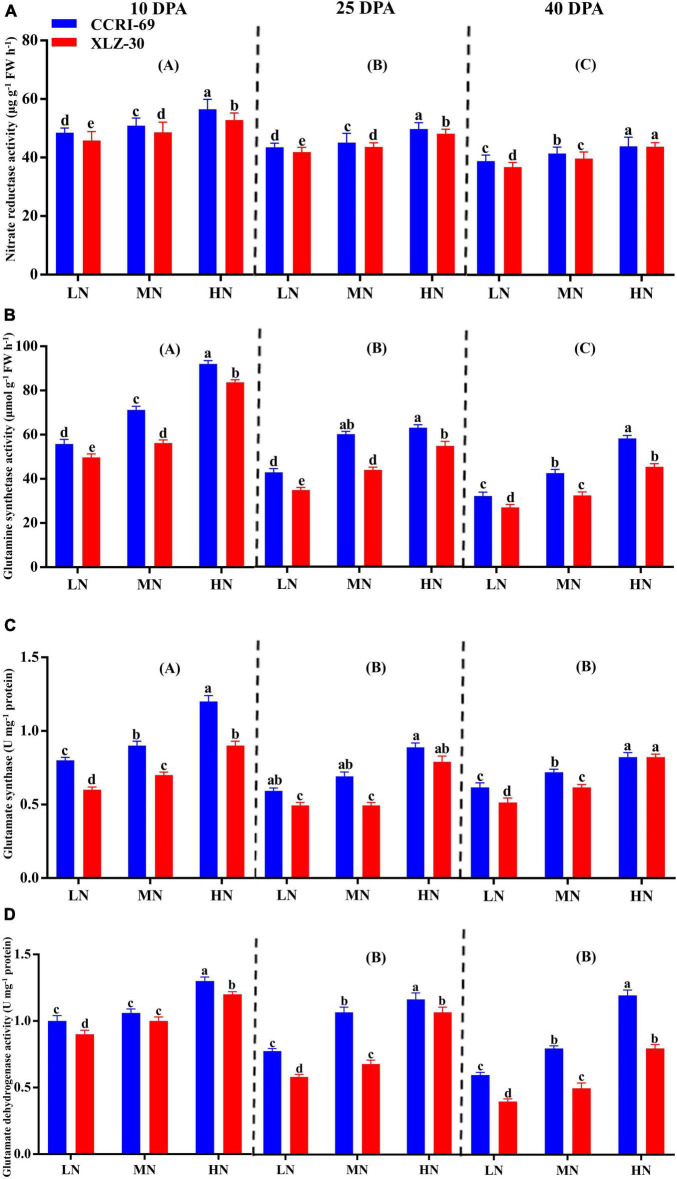
**(A–D)** Nitrate reductase activity (μg g^–1^ FW h^–1^), glutamine synthetase activity (μmol g^–1^ FW h^–1^), glutamate synthase (U mg^–1^ protein), and glutamate dehydrogenase activity (U mg^–1^ protein) in cotton subtending leaves of CCRI-69 and XLZ-30 in response to low (LN; 0.25 mM), moderate (MN; 2.5 mM), and high (HN; 5 mM) N levels at 10, 25, and 40 days post-anthesis (DPA). Error bars with different small letters show a significant difference between genotypes under different N levels and capital letters in the brackets show significant differences among growth stages (10, 25, and 40 DPA) at *p* < 0.05.

A significant reduction in N metabolizing enzymatic activities was observed in XLZ-30 under low N level from 10 to 40 DPA (Figure 3A). In comparison with low N level, high N level increased NR activity by 13.6, 12.8, and 13.8%, GS activity by 39.9, 34.2, and 43.0%, GOGAT activity by 32.3, 33.8, and 33.4%, and GDH activity by 18.8, 40.1, and 50.2% at 10, 25, and 40 DPA, respectively (Figure 3). However, the GOGAT and GDH activities at 25 and 40 DPA were statistically similar but lower than that of 10 DPA. Irrespective of the N levels, enzymatic activities like NR (5.6, 3.5, and 3.2%), GS (13.4, 19.7, and 21.2%), GOGAT (20.1, 22.2, and 8.5%), and GDH (11.6, 24.2, and 29.7%) increased in the subtending leaf of CCRI-69 at 10, 25, and 40 DPA, respectively, as compared to XLZ-30 (Figure 3).

**FIGURE 4:**
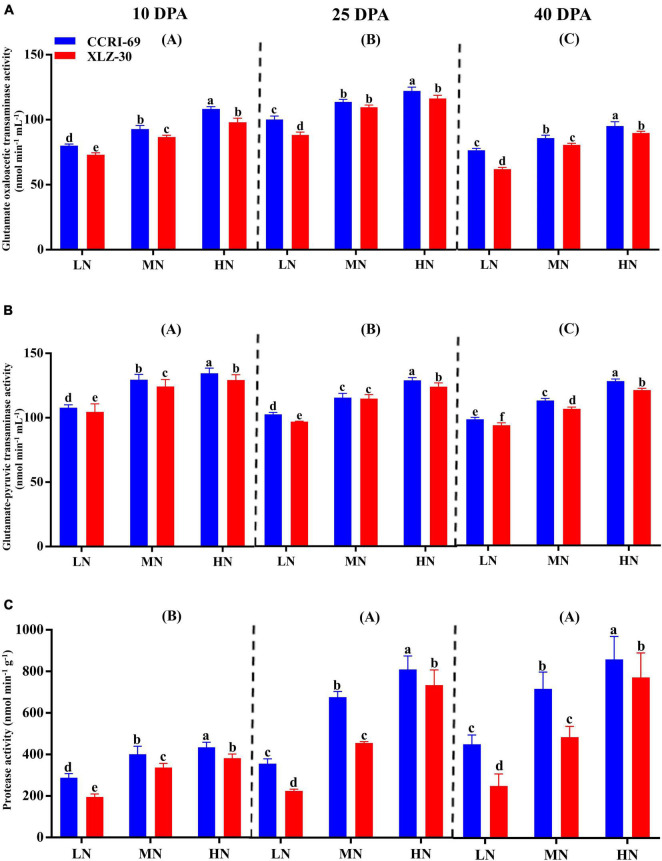
**(A–C)** Glutamate oxaloacetic transaminase activity (nmol min^–1^ mL^–1^), glutamate–pyruvic transaminase activity (nmol min^–1^ mL^–1^), and protease activity (nmol min^–1^ g^–1^) in cotton subtending leaves of CCRI-69 and XLZ-30 in response to low (LN; 0.25 mM), moderate (MN; 2.5 mM), and high (HN; 5 mM) N levels on 10, 25, and 40 days post-anthesis (DPA). Error bars with different small letters show a significant difference between genotypes under different N levels, and capital letters in the brackets show significant differences among growth stages (10, 25, and 40 DPA) at *p* < 0.05.

The GOT and GPT activities consistently decreased from 10 to 40 DPA (Figures 4A,B). The peak activities were observed under high N level where GOT increased by 25.8, 25.2, and 20.9%, and GPT by 19.5, 21.2, and 22.9% at 10, 25, and 40 DPA, respectively, as compared to low N level (Figures 4A,B). Compared to XLZ-30, the average GOT and GPT activities in the subtending leaf of CCRI-69 increased across N levels with 8.4 and 3.7% at 10 DPA, 9.8 and 3.3% at 25 DPA, and 6.5 and 5.3% at 40 DPA, respectively (Figures 4A,B). A clear increase in the protease activity was observed from 10 DPA to 40 DPA (Figure 4C). Application of high N level increased the protease activity by 40.8, 62.4, and 57.0% at 10, 25, and 40 DPA, respectively, as compared to low N level (Figure 4C). In comparison with XLZ-30, protease activity in the subtending leaf of CCRI-69 was increased by 18.6, 23.2, and 25.6% at 10, 25, and 40 DPA, respectively (Figure 4C). At each N level, the higher protease activity in the subtending leaf of CCRI-69 indicates the high protein degradation and more N remobilization to the sink during boll development.

Sucrose phosphate synthase, SS, and sucrose content in the cotton subtending leaf were declined from 10 to 40 DPA in both cotton genotypes, and the lowest values were recorded at 40 DPA (Supplementary Figure 2). A clear decline in SPS activity was recorded from 10 to 40 DPA, with a more reduction at 40 DPA (Supplementary Figure 2A). Under a high N level, the SPS activity increased 51.0, 52.5, and 58.7% at 10, 25, and 40 DPA as compared to low N level, respectively (Supplementary Figure 2A). Irrespective of the N levels, the increase of SPS activity in the subtending leaf of CCRI-69 was 12.6% at 10 DPA, 14.6% at 25 DPA, and 18.8% at 40 DPA as compared to XLZ-30 (Supplementary Figure 2A. The SS activity in cotton subtending leaves of both cotton genotypes was increased at the rate of 51.7, 52.4, and 26.3% at 10, 25, and 40 DPA, respectively (Supplementary Figure 2B). In comparison with XLZ-30, SS activity in the subtending leaf of CCRI-69 was 9.6, 8.3, and 6.6% higher at 10, 25, and 40 DPA, respectively (Supplementary Figure 2B). The sucrose contents in the leaves of both cotton genotypes were increased under a high N level, however, the increase greatly varied among the growth stages, where 40.4% at 10 DPA, 40.8% at 25 DPA, and 47.5% at 40 DPA were recorded (Supplementary Figure 2C). Meanwhile, the increase in subtending leaf sucrose content of CCRI-69 was 11.4% at each 10 and 25 DPA, and 13.8% at 40 DPA as compared to XLZ-30 (Supplementary Figure 2C). Moreover, the variation in cotton genotypes in response to N levels was less at 40 DPA, whereas no significant difference was noted at high N level, however, subtending leaf sucrose content of XLZ-30 at moderate were similar to that of CCRI-69 at low N level (Supplementary Figure 2C).

### Nitrogen Concentration and Use Efficiency

**FIGURE 5:**
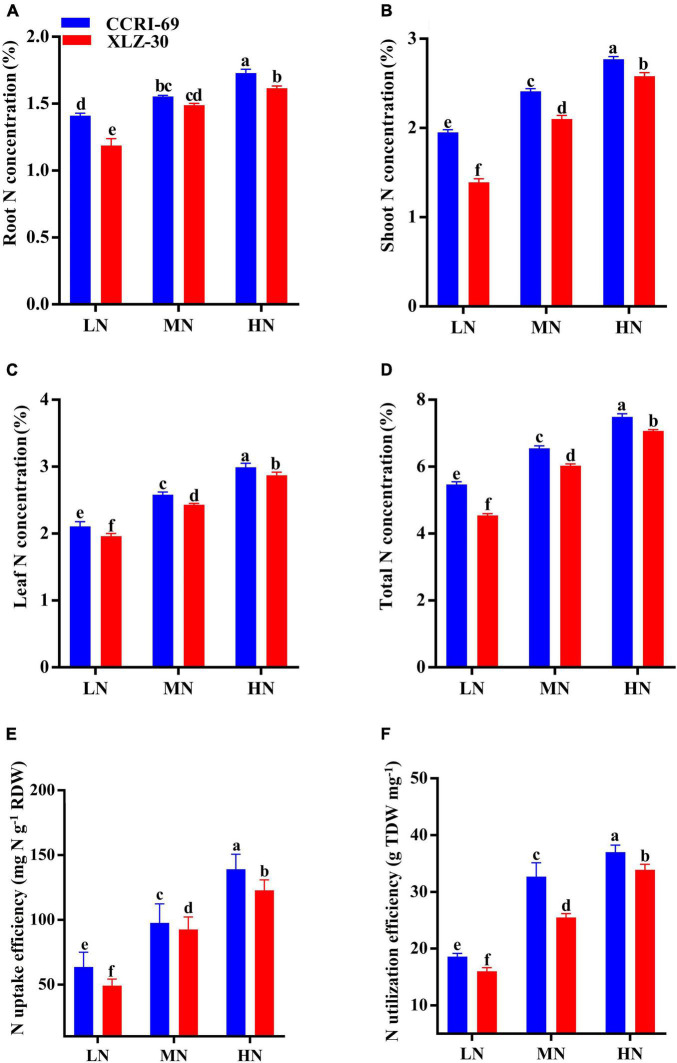
**(A–F)** Root N concentration (%), shoot N concentration (%), leaf N concentration (%), total plant N concentration (%), NUpE (mg N g^–1^ RDW), and NUtE (g TDW mg^–1^) of CCRI-69 and XLZ-30 in response to low (LN; 0.25 mM), moderate (MN; 2.5 mM), and high (HN; 5 mM) N levels. Error bars with different small letters show significant differences between genotypes under different N levels (*p* < 0.05).

Nitrogen concentration of root, shoot, leaf, and total N concentration abruptly dropped under low N level, with an approximate decrease of 23.3, 37.4, 30.6, and 21.3%, respectively (Figures 5A–D). In comparison with XLZ-30, N concentration in CCRI-69 increased by 12.4% on an average in the total plant (Figures 5A–D). The NUpE increased by 56.9% under a high N level as compared to the low N level. Irrespective of the N levels, NUpE in the CCRI-69 was 11.9% higher than XLZ-30 (Figure 5E). Similarly, NUtE also increased with an increase in N levels, and a significant increase of 48.3% was observed under high N level as compared to low N level. Average across the N levels, NUtE in CCRI-69 increased by 12.3% than XLZ-30 (Figure 5F).

### Yield and Yield Attributes

**FIGURE 6:**
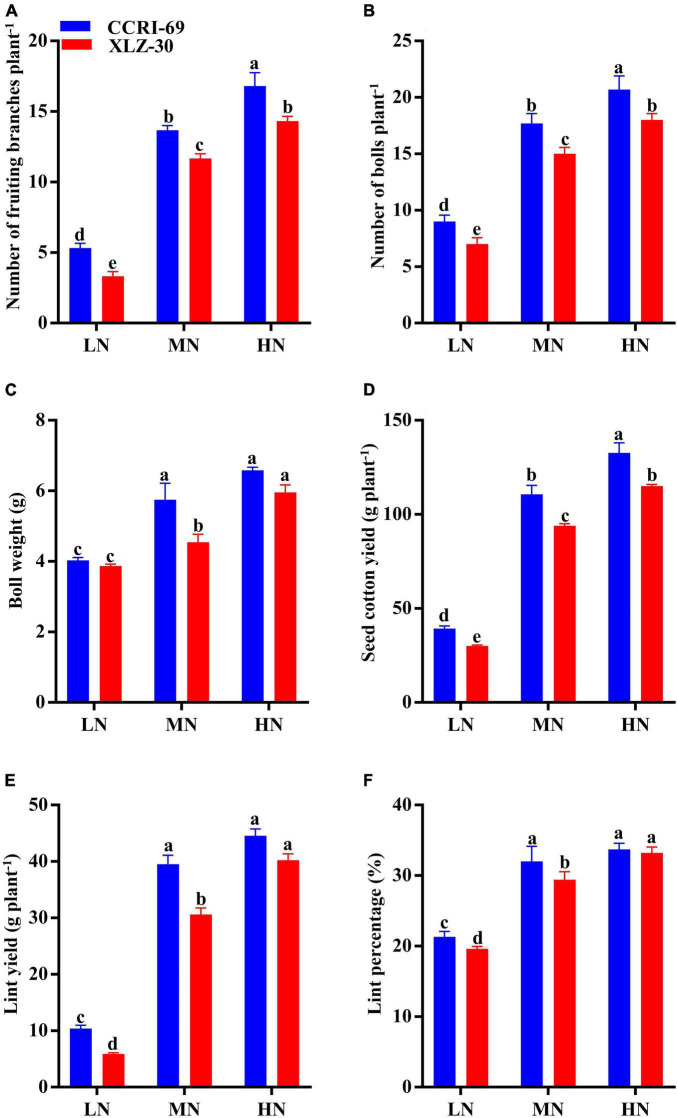
**(A–F)** Number of fruiting branches plant^–1^, number of bolls plant^–1^, boll weight (g), seed cotton yield (g plant^–1^), lint yield (g plant^–1^), and lint percentage (%) of CCRI-69 and XLZ-30 in response to low (LN; 0.25 mM), moderate (MN; 2.5 mM), and high (HN; 5 mM) N levels. Error bars with different small letters show significant differences between genotypes under different N levels (*p* < 0.05).

The yield and yield attributes of both cotton genotypes were significantly influenced by N levels (Figure 6). Application of high N level increased the number of fruiting branches, number of bolls plant^–1^, boll weight, seed cotton yield, lint yield, and lint percentage by 72.2, 58.6, 37.0, 74.0, 82.7, and 38.9%, respectively, as compared to low N level (Figure 6). A clear genotypic difference was observed for yield and yield attributes, where CCRI-69 has a higher number of fruiting branches (18.1%), number of bolls plant^–1^ (15.5%), boll weight (12.2%) seed cotton yield (26.1%), lint yield (18.6%), and lint percentage as compared to XLZ-30 (Figure 6). Moreover, the yield and yield components of XLZ-30 at a high N level were similar to that of CCRI-69 at a moderate N level, indicating the high N efficiency of CCRI-69 at relatively reduced N fertilization.

### Relationships Between Nitrogen-Nutrition Status in the Subtending Leaf With Carbon and Nitrogen Metabolism and Yield

The photosynthesis and C/N metabolism in the cotton subtending leaf can indicate the source strength and are closely related to the leaf N-nutrition status. Therefore, if the relationship between the leaf N status and C/N metabolism of contrasting N-efficient cotton genotypes can be clarified, the leaf source strength and the efficiency of a genotype could be easily evaluated on a real-time basis and the yield and quality of the cotton could be predicted using the leaf N status.

**FIGURE 7:**
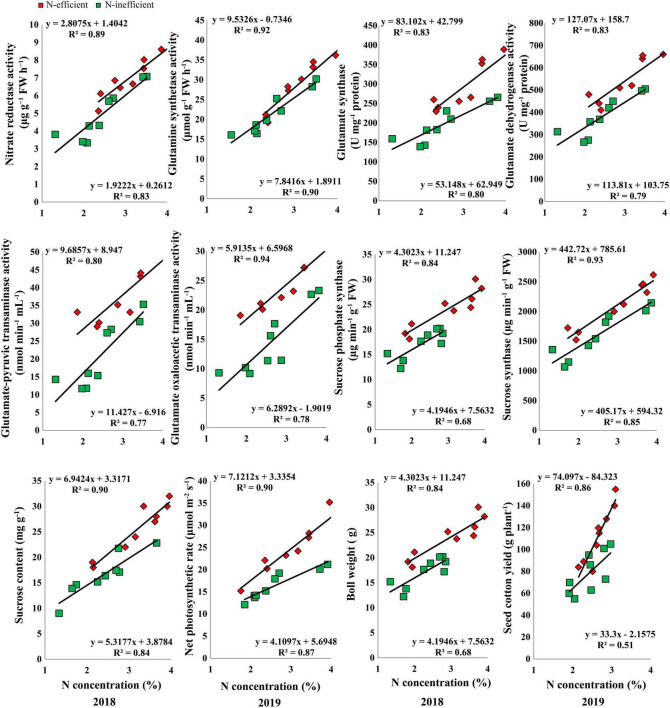
Relationships of subtending leaf N concentration (%) with nitrate reductase (μg g^–1^ FW h^–1^), glutamine synthetase activity (μmol g^–1^ FW h^–1^), glutamate synthase (U mg^–1^ protein) and dehydrogenase activity (U mg^–1^ protein), glutamate–pyruvic transaminase activity (nmol min^–1^ mL^–1^), glutamate oxaloacetic transaminase activity (nmol min^–1^ mL^–1^), sucrose phosphate synthase (μg min^–1^ g^–1^ FW), sucrose synthase (μg min^–1^ g^–1^ FW), sucrose content (mg g^–1^ FW), net photosynthetic rate (μmol m^–2^ s^–1^), boll weight (g), and seed cotton yield (g plant^–1^) of N-efficient (CCRI-69) and N-inefficient (XLZ-30) cotton genotypes under hydroponic condition.

The results of the hydroponic experiment revealed that N concentration in the subtending leaf of the contrasting N-efficient (CCRI-69, N-efficient and XLZ-30, N-inefficient) cotton genotypes showed a positive linear relationship with key enzymes related to C/N metabolism, sucrose content, net photosynthetic rate, boll weight, and seed cotton yield (Figure 7). The slope of the fitted line was slightly steeper for GS, GOT, and net photosynthetic rate between N-efficient and N-inefficient cotton genotypes. However, the slope of the NR, GOGAT, GDH, GPT, SPS, SS, sucrose content, boll weight, and seed cotton yield were comparatively steeper in N-inefficient than N-efficient cotton genotypes (Figure 7).

**FIGURE 8:**
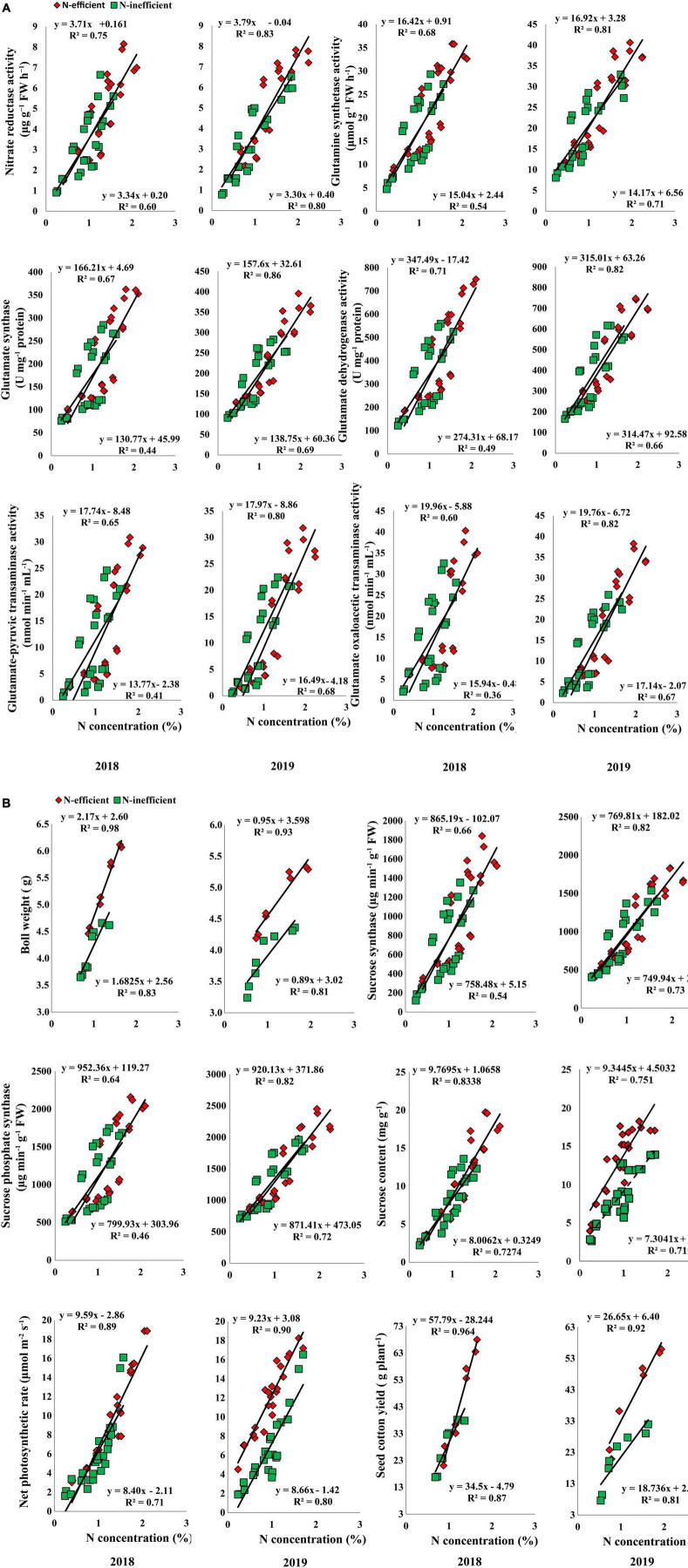
**(A)** Relationships of subtending leaf N concentration (%) with nitrate reductase (μg g^–1^ FW h^–1^), glutamine synthetase activity (μmol g^–1^ FW h^–1^), glutamate synthase (U mg^–1^ protein), and glutamate dehydrogenase activity (U mg^–1^ protein), glutamate–pyruvic transaminase activity (nmol min^–1^ mL^–1^), glutamate oxaloacetic transaminase activity (nmol min^–1^ mL^–1^) of N-efficient (CCRI-69) and N-inefficient (XLZ-30) cotton genotypes under pot condition in 2018 and 2019. **(B)** Relationships of subtending leaf N concentration (%) with boll weight (g), sucrose synthase (μg min^–1^ g^–1^ FW), sucrose phosphate synthase (μg min^–1^ g^–1^ FW), sucrose content (mg g^–1^ FW), net photosynthetic rate (μmol m^–2^ s^–1^), and seed cotton yield (g plant^–1^) of N-efficient (CCRI-69) and N-inefficient (XLZ-30) cotton genotypes under pot condition in 2018 and 2019.

In the pot experiment, N concentration in the subtending leaf showed a stronger relationship with NR, GS, GOGAT, GDH, GOT, and GPT in 2018. In 2019, the slope of the fitted line of NR was similar for both types of cotton genotypes. However, the slope of GS, GOGAT, GDH, GOT, and GPT was comparatively steeper between N-inefficient and N-efficient cotton genotypes (Figure 8A). Similarly, a strong positive relationship was also noted for boll weight, SS, SPS, sucrose content, net photosynthetic rate, and seed cotton yield in 2018. Except for sucrose content, the slope of the boll weight, SS, SPS, net photosynthetic rate, and seed cotton yield with subtending leaf N concentration were steeper for N-inefficient genotype as compared to the N-efficient cotton genotype in 2019 (Figure 8B).

**FIGURE 9:**
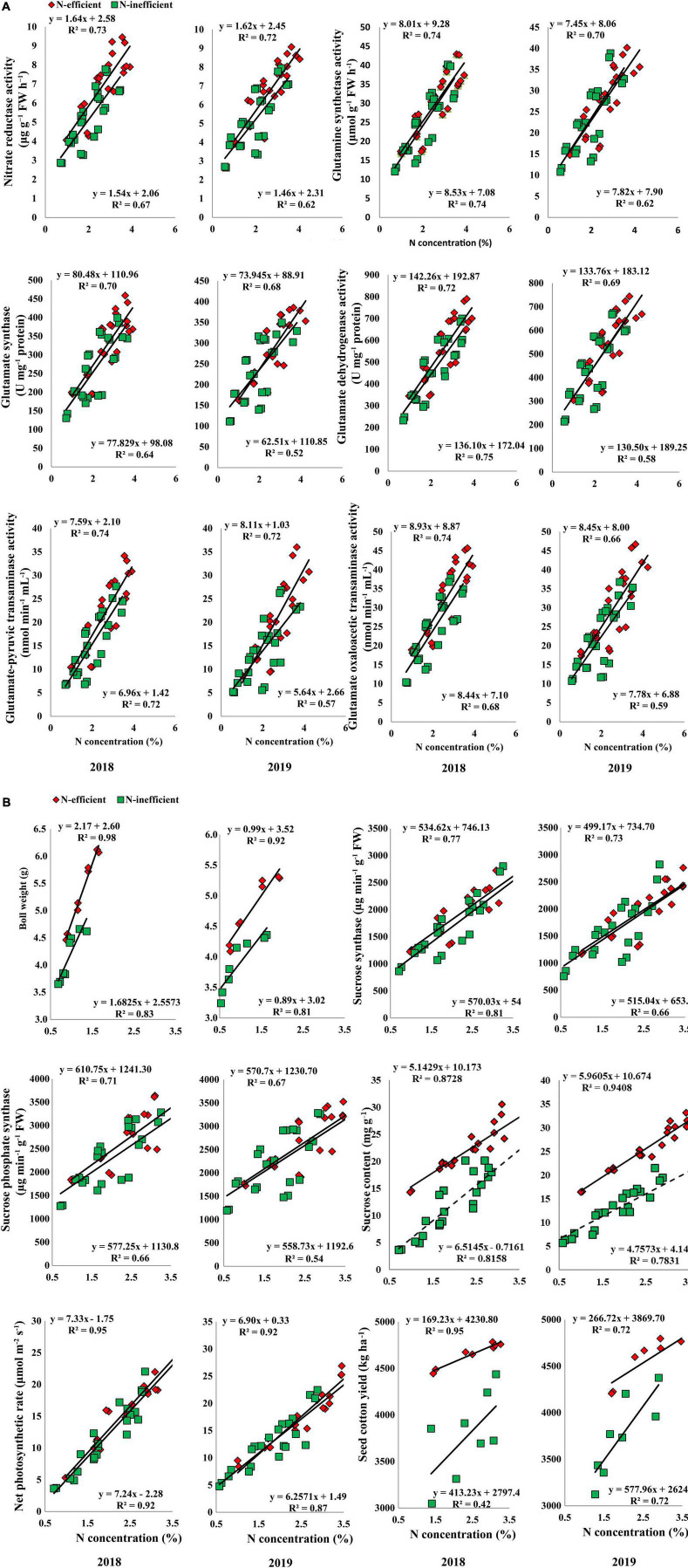
**(A)** Relationships of subtending leaf N concentration (%) with nitrate reductase (μg g^–1^ FW h^–1^), glutamine synthetase activity (μmol g^–1^ FW h^–1^), glutamate synthase (U mg^–1^ protein), and glutamate dehydrogenase activity (U mg^–1^ protein), glutamate-pyruvic transaminase activity (nmol min^–1^ mL^–1^), glutamate oxaloacetic transaminase activity (nmol min^–1^ mL^–1^) of N-efficient (CCRI-69), and N-inefficient (XLZ-30) cotton genotypes under field condition in 2018 and 2019. **(B)** Relationships of subtending leaf N concentration (%) with boll weight (g), sucrose synthase (μg min^–1^ g^–1^ FW), sucrose phosphate synthase (μg min^–1^ g^–1^ FW), sucrose content (mg g^–1^ FW), net photosynthetic rate (μmol m^–2^ s^–1^), and seed cotton yield (kg ha^–1^) of N-efficient (CCRI-69) and N-inefficient (XLZ-30) cotton genotypes under field condition in 2018 and 2019.

A strong positive relationship was also observed for the selected traits in the field experiment under both cropping seasons. The slope of the fitted line between subtending leaf N concentration and GS, GDH, and GPT was almost similar; however, the slope of NR, GOGAT, and GOT was comparatively steeper between N-inefficient and N-efficient genotypes in 2018. The slope between subtending leaf N concentration and NR, GS, GOGAT, GDH, GPT, and GOT was steeper for N-inefficient cotton genotype as compared to N-efficient in 2019 (Figure 9A). Notably, subtending leaf N concentration has a strong positive relationship with boll weight, SS, SPS, sucrose content, net photosynthetic rate, and seed cotton yield in 2018. Similarly, in 2019, subtending leaf N concentration has a strong positive relationship with boll weight, SS, SPS, sucrose, net photosynthetic rate, and seed cotton yield. In comparison with 2019, the slope between subtending leaf N concentration and seed cotton yield of N-inefficient genotype was steeper than N-efficient genotype in 2018 (Figure 9B).

Overall, N concentration in the subtending leaf of N-efficient cotton genotype showed a strong relationship with C/N metabolizing enzymes, net photosynthetic rate, sucrose contents, boll weight, and seed cotton yield as compared to N-inefficient genotype in the hydroponic, pot, and field conditions. These results indicate that N concentration in the subtending leaf regulates C/N metabolism, supports high photosynthesis, sucrose production, and finally cotton yield.

## Discussion

Due to an indeterminate growth habit, cotton plants show several morphological and physiological adaptations to environmental conditions, such as genotype and N levels (Iqbal et al., 2020a). N is one of the most important nutrients needed in large amounts for better cotton production (Iqbal et al., 2020a). The application of optimum N improves crop growth and production. However, suboptimal N application results in poor crop productivity due to the downregulation of important physiological processes such as photosynthesis and N metabolism (Iqbal et al., 2020b). In the present study, dynamics of growth, photosynthetic attributes, and C/N metabolism of cotton subtending leaves were studied in relation to cotton yield in response to different N levels. A better sink (reproductive parts) development has a close relationship with leaf morphology and physiology (Hu et al., 2015), and a well-established leaf area is the guarantee of high photosynthates production in the plants. A considerable variation was observed in various growth attributes in cotton genotypes in response to N levels. Across the average of N levels, CCRI-69 had comparatively higher growth, indicating that CCRI-69 can perform better at a relatively low N level. Moreover, growth attributes, like root, shoot, and total plant dry matter, shoot length, and leaf area plant^–1^ increased in CCRI-69 as the N level increased, which was consistent with the previous findings (Luo et al., 2015; Iqbal et al., 2020a,b). As mentioned earlier that high leaf area is the source for more photosynthates production which is partitioned into reproductive parts for supporting high yield and yield components. This statement is supported by the strong positive correlation between subtending leaf N concentration with net photosynthetic rate, sucrose contents, and seed cotton yield and also by the previous studies that have found that high leaf area is important for the development of boll and cotton yield (Xu et al., 2015; Qin et al., 2018).

Photosynthesis is closely associated with N uptake and availability in cotton (Iqbal et al., 2020b). It is the most essential process for plant growth and development (Hafeez et al., 2019), and it is rigorously affected by low N availability in cotton because it is more sensitive to N in comparison with other crops (Iqbal et al., 2020a). This is because 57% of the leaf N is located in the chloroplast and the enzymes involved in photosynthesis (Ziadi et al., 2008; Xu et al., 2012). Compared to low N, moderate to high N levels improved net photosynthetic rate in the subtending leaves of CCRI-69 across the growth stages (Figure 2A), indicating high photosynthetic efficiency in CCRI-69, which supports higher yield. The increase in yield might be due to the higher translocation of photoassimilates from subtending leaf to reproductive parts (Chen et al., 2019). Similarly, our earlier study also showed that CCRI-69 had higher photosynthetic efficiency than XLZ-30 at the seedling stage (Iqbal et al., 2020a,b). The reduction in photosynthesis in the subtending leaf of XLZ-30 might be due to low leaf area and poor photosynthetic activity due to the downregulation of the genes responsible for photosynthetic pathways observed in the transcriptomic study (data not published). The poor photosynthesis in XLZ-30 may also be due to lower carboxylation rate, as shown by the high intercellular CO_2_ concentration (Figure 2C), which is consistent with the results obtained from rice as well as in our previous study in cotton (Huang et al., 2004; Iqbal et al., 2020b). Similarly, the biochemical factors controlling photosynthesis like N metabolizing enzymatic activities within the plant leaves were also greatly influenced by low N (Iqbal et al., 2020a,b), following poor translocation of photoassimilates into the reproductive parts, causing a feedback inhibition of photosynthesis. Thus, reduction in subtending leaf photosynthetic activity at the low N level, especially in XLZ-30, may be the consequence of declined physiological processes involved in photosynthesis.

In the current study, cotton yield and yield components of both cotton genotypes were enhanced in response to an increase in N levels, with more increase in CCRI-69 (Figure 6). These results suggest that N plays a vital role in the various physiological processes like photosynthesis in the plants which maintains optimum plant growth and productivity (Iqbal et al., 2020a). The higher cotton yield could be attributed to better N assimilation and photoassimilates translocation from subtending leaf to reproductive organs. A similar explanation was reported for K application by Ali et al. (2018), and Iqbal et al. (2020a,b) for N in cotton. The increase in the number of bolls plant^–1^ and boll weight in moderate to high N-treated plants indicate that these yield components are directly affected by N fertilization in cotton (Figures 6B,C). In consistent with our results, Chen et al. (2019) also suggested that moderate N fertilization in field-grown cotton is better for higher yield and yield components. In contrast, low or lack of N fertilization inhibited boll and yield formation because boll development is the process of storage and redistribution of photoassimilates as well as the synthesis of various metabolites, which are constrained by low N (Boquet and Breitenbeck, 2000). In a similar pattern, low N-treated plants have poor photosynthesis and photosynthates translocation to the boll, which resulted in lesser number of bolls plant^–1^, low boll weight, and hence low yield as compared to moderate and high N levels (Bondada and Oosterhuis, 2001). In addition, the N contents in the shells, seeds, and physiological metabolism will rise (Ferrari et al., 2011), which would inhibit the transportation of photoassimilates to the boll and eventually decrease the boll weight and cotton yield under low N level (Chen et al., 2019). Thus, the increase in boll number and weight under moderate to high N levels in CCRI-69 could be attributed to higher photosynthetic efficiency and more photosynthates translocation, which encourages the development of better reproductive organs and hence yield.

Nitrogen-efficient cotton genotype (CCRI-69) had a significantly higher yield and NUE (Figures 5, 6). This yield gain can be associated with comparatively higher N metabolizing enzymatic activities (NR, GS, GOGAT, GDH, GOT, GPT, and protease), and high levels of each promoted number of bolls, boll weight, and yield formation. During the reproductive stage, most of the N is remobilized from leaves to the developing bolls by the hydrolysis of proteins to amino acids through protease activity (Schrader and Thomas, 1981). This selective dry matter partitioning to reproductive than vegetative organs under moderate to high N levels could promote yield in cotton. Studies have found that leaves are the most effective site of N metabolism. Therefore, a large amount of N is translocated to the leaves soon after the uptake for assimilation (Iqbal et al., 2020a) into different amino acids, proteins, and N-containing compounds (Pratelli and Pilot, 2014) through various enzymatic activities (Hakeem et al., 2011; Funayama et al., 2013). Among various N metabolizing enzymes, NR is the principal and one of the most rate-limiting enzymes in N metabolism (Mokhele et al., 2012). Similarly, GS also plays a very crucial role in N metabolism, depending upon the extent of N assimilation, growth stage (Mokhele et al., 2012; Hildebrandt et al., 2015), NR activity, and the N supply. After uptake, nitrate is converted into nitrite and then ammonia *via* NR and NiR (Cao et al., 2012). The ammonia produced is then converted into glutamine and glutamate by GS and GOGAT, respectively, to form various amino acids. The amino acids produced from GS/GOGAT cycle are then used for the synthesis of protein in the leaves (Wickert et al., 2007; Mokhele et al., 2012) during primary N assimilation (Pratelli and Pilot, 2014). Glutamic acid produced during the primary N assimilation is used for the synthesis of aspartic acid and glutathione *via* GOT and GPT enzymes, respectively. In addition, glutamine, glutamate, aspartate, and asparagine are also the primary products of N assimilation in the leaves (Coruzzi, 2003). The extent of these amino acids in the leaves of plants changes from time to time due to protein synthesis and degradation or changes in N enzymatic activities like protease (Hildebrandt et al., 2015), as observed in the current study (Figure 4). GDH is another essential N metabolizing enzyme working as a shunt between C/N metabolism, and its activity increases when plants are under stress conditions, especially N stress. It catalyzes the amination of 2-oxoglutarate to glutamate and the deamination of glutamate to 2-oxoglutarate (Miyashita and Good, 2008; Mokhele et al., 2012). In the current study, the plants treated with high N level improved the activities of enzymes to efficiently regulate N metabolism in the cotton subtending leaves (Figure 3D). Comparatively, the N assimilating enzymatic activities were high in the subtending leaf of CCRI-69 as compared to XLZ-30 in response to various N levels across all the growth stages (Figures 3, 4). The increase in enzymatic activities in CCRI-69 is consistent with the results obtained from the N-efficient *Brassica napus* genotype as well as in Arabidopsis plants (Lemaître et al., 2008; Ye et al., 2010). The high enzymatic activities in the CCRI-69 might be associated with the upregulation of N transporters (data unpublished) and the genes responsible for these enzymes, as shown in our previous study (Iqbal et al., 2020c). A similar assumption was reported for finger millet genotypes based on Arabidopsis nitrate transporters (Gupta et al., 2012). Moreover, the high N metabolism in CCRI-69 might be due to the continuous conversion of nitrate to N-containing compounds, as mentioned in the previous reports (Ali et al., 2007; Vijayalakshmi et al., 2015). Thus, high NUE and yield of CCRI-69 are due to a well-organized system of N uptake, transport, N metabolism (Yun et al., 2008), protein degradation, and remobilization following higher photosynthates partitioning to sink tissues.

## Conclusion

Nitrogen plays a crucial role in growth, photosynthesis, C/N metabolism as well as in cotton yield. This role is complex and varied in cotton genotypes, as it involvs several physiological processes. Growth, photosynthetic attributes, and sucrose content in the subtending leaves of cotton genotypes, especially CCRI-69, were substantially influenced by N levels across the growth stages. Moreover, higher N application not only managed C/N metabolism in cotton subtending leaves of CCRI-69, but also directed their best use within the plant. The activities of various C/N metabolizing enzymes such as NR, GS, GOGAT, GDH, GPT, GOT, protease, SPS, and SS were increased by moderate to high N levels as compared to low N. Overall, the growth, photosynthesis, sucrose content, C/N metabolizing enzymes, yield, and yield components of XLZ-30 at a high N level were similar to that of CCRI-69 at a moderate N level. Thus, comparatively low N is sufficient for CCRI-69 to improve growth, photosynthesis, and balance C/N metabolism in the cotton subtending leaf to achieve a high yield. Moreover, this study exhibits the significance of N-efficient cotton genotype, which can be grown with limited N supply for environment-friendly farming systems, thus reducing the load of N on the soil.

## Data Availability Statement

The raw data supporting the conclusions of this article will be made available by the authors, without undue reservation.

## Author Contributions

SM, AI, and DQ: conceptualization. AI, NJ, ZH, and WX: data curation. GH and ZH: formal analysis. SM and ZX: funding acquisition. AI, NJ, and PN: investigation. AI, DQ, and GH: methodology. SM, ZX, and DQ: project administration and supervision. SM, ZX, WX, and DQ: resources and software. AI and SM: validation. AI, SM, and WX: visualization. AI and DQ: writing – original draft. AI, SM, ZX, and DQ: writing – review and editing. All authors contributed to the article and approved the submitted version.

## Conflict of Interest

The authors declare that the research was conducted in the absence of any commercial or financial relationships that could be construed as a potential conflict of interest.

## Publisher’s Note

All claims expressed in this article are solely those of the authors and do not necessarily represent those of their affiliated organizations, or those of the publisher, the editors and the reviewers. Any product that may be evaluated in this article, or claim that may be made by its manufacturer, is not guaranteed or endorsed by the publisher.
