# Supplementary material for: Physiological Characteristics of Cotton Subtending Leaf Are Associated With Yield in Contrasting Nitrogen-Efficient Cotton Genotypes
Source: Front Plant Sci. 2022 Feb 7;13:825116. doi: 10.3389/fpls.2022.825116 (PMC8859460; doi:10.3389/fpls.2022.825116)
Supplement: Supplementary file 1 [file Table_1.DOCX]

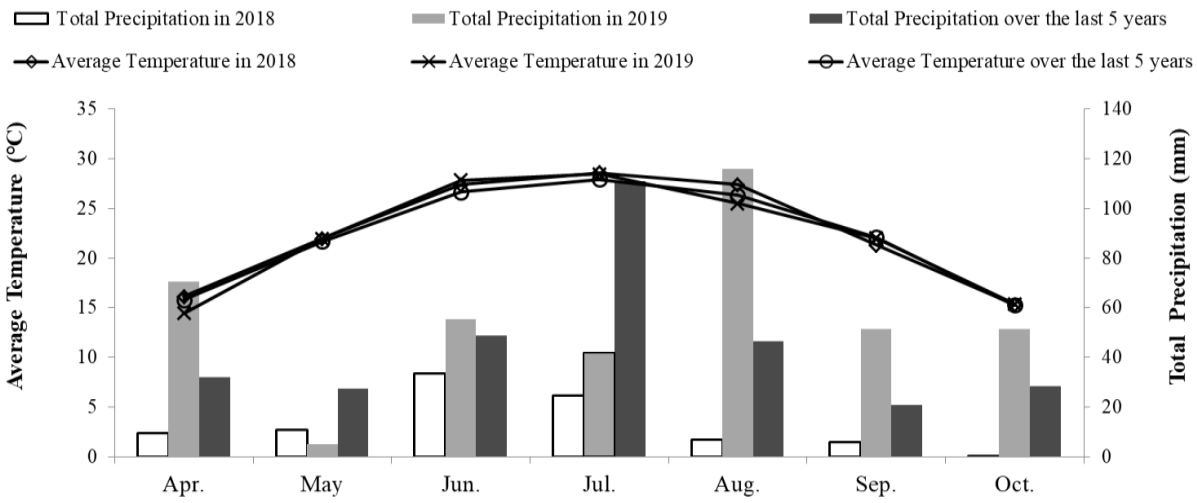
Figure S1. Meteorological data during the cotton growth period of the field experiment from May to October during 2018 and 2019 in Anyang, China.


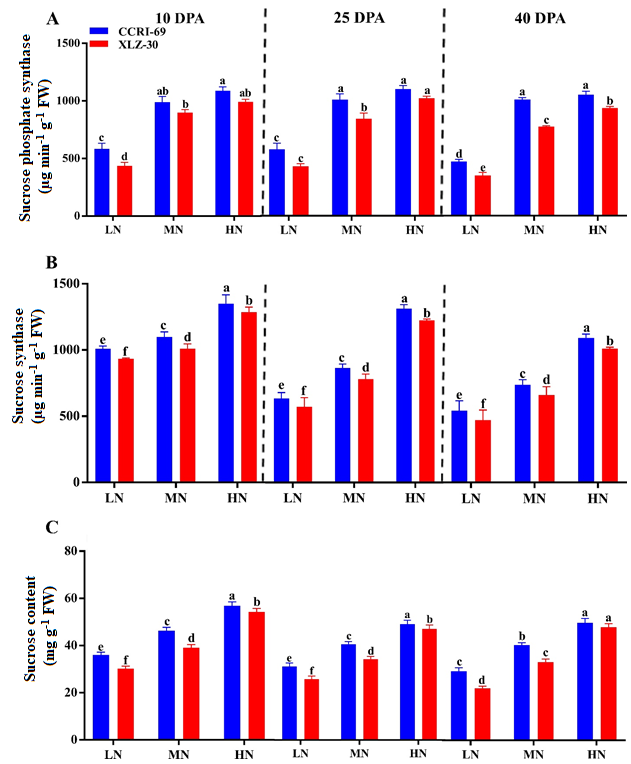


Figure S2. (A) Sucrose phosphate synthase (SPS; µg min^-1^ g^-1^ FW), (B) sucrose synthase (SS; µg min^-1^ g^-1^ FW), and (C) sucrose content (mg g^-1^ FW) in the cotton subtending leaves of CCRI-69 and XLZ-30 in response to low (LN; 0.25 mM), moderate (MN; 2.5 mM), and high (HN; 5 mM) N levels at 10, 25, and 40 days post-anthesis (DPA). Error bars with different small letters show a significant difference between genotypes under different N levels at p < 0.05.
